# Psychotherapists' Knowledge of Guideline Recommendations for the Treatment of Depressed Suicidal Patients

**DOI:** 10.3389/fpsyt.2022.873127

**Published:** 2022-04-14

**Authors:** Tobias Teismann, Helena Düwel, Leandra Eidt, Julia Brailovskaia, Jan Christopher Cwik

**Affiliations:** ^1^Mental Health Research and Treatment Center, Ruhr-Universität Bochum, Bochum, Germany; ^2^Department of Clinical Psychology and Psychotherapy, Universität zu Köln, Cologne, Germany

**Keywords:** suicide, suicide attempt, guideline, treatment, adherence

## Abstract

**Objective:**

Clinical practice guidelines present expert consensus on the treatment of mental disorders. Yet, studies have shown that knowledge of and adherence to recommendations are moderate. The aim of the present study was to investigate, whether and to what extent psychotherapists are aware of and follow the German guideline recommendations for the treatment of suicidal depressed patients.

**Methods:**

174 participants (licensed psychotherapists, psychotherapists-in-training) were presented with five groups of guideline recommendations (referring to inpatient admission, psychotherapy, acute pharmacotherapy, pharmacologic relapse prevention, follow-up appointments) and were asked to identify the guideline recommendation and indicate whether they provided treatment according to the guideline.

**Results:**

Knowledge and adherence to the guidelines recommendations on psychotherapy and inpatient admission were well present. However, knowledge about pharmacological treatment recommendations was low; same as the knowledge on the necessity of immediate follow-up appointments after discharge of patients hospitalized due to suicidality.

**Discussion:**

The results highlight the importance of greater dissemination of various facts about the management of suicidal patients.

## Introduction

Clinical practice guidelines present expert consensus on how mental disorders should be diagnosed and treated. On the one hand, recommendations are made for interventions that are evidence-based and effective when the risk-benefit profile is weighed up; on the other hand, interventions are discouraged for which studies have failed to provide evidence of effectiveness or for which ineffectiveness or even a negative effect has been demonstrated. Guidelines have been formulated and implemented in many countries. In Germany, a large number of guidelines exists for the treatment of a wide variety of physical illnesses and mental disorders (www.awmf.org). Recommendations made in guidelines are not legally binding, i.e., clinicians may deviate from treatments recommended in the guideline; however, treatment in accordance with the guideline offers potential protection against legal disputes. Furthermore, the implementation of evidence-based guidelines is expected to improve the quality of pharmacological and psychotherapeutic care of people with mental illnesses (1, 2).

In Germany there is so far no dedicated guideline for the management of suicidality in adults (3). There is a guideline for the treatment of suicidal ideation and behavior in childhood and adolescence (4), and guidelines for the treatment of various disorders (including bipolar disorder, schizophrenia, depression) contain chapters on dealing with suicidality. Against the background of the close association between depression and suicidality (5) it is not surprising that the national guideline on the treatment of unipolar depression (6) provides comprehensive and specific guidance on risk assessment, inpatient admission, psychotherapy, and pharmacotherapy for suicidal depressed patients (www.leitlinien.de/themen/depression/2-auflage). The chapter on the management of suicidality from the German guideline for the treatment of unipolar depression thus currently bundles the most important guidelines for dealing with suicidal patients in Germany. However, it is unknown in as much mental health professionals adhere to these specific guideline recommendations.

In general, studies have documented low rates of adherence to guideline recommendations (7–10): with regard to the treatment of depressed patients, adherence rates of about 50% were found in various studies (1, 9–12). In a study on adherence to depression guidelines in primary care settings, Hepner et al. (1) reported especially low adherence rates regarding suicidality management: “Suicidality usually was not assessed (adherence, 24%), was not treated with appropriate medications or psychotherapy (adherence, 26%), and did not result in mental health specialist consultation (adherence, 36%)” (p. 324). A lack of awareness and a lack of familiarity have been shown to be important reasons for non-adherence to guideline recommendations (13).

On this background, the aim of the current study was to investigate, whether and to what extent German psychotherapists are aware of and follow the guideline recommendations for the treatment of suicidal depressed patients (6). For this purpose, participants were presented with an array of multiple-choice questions containing the correct guideline recommendations as well as incorrect/distraction answers. In addition, psychotherapists were asked whether they adhered to the non-pharmacological recommendations in their clinical practice. This is an exploratory study, so no a-priori hypotheses have been formulated.

## Materials and Methods

### Participants

In total, 607 persons entered the study website, 292 persons read the study instruction and 200 persons took part in the study. Twenty-six participants had to be excluded since they were neither psychotherapists in training nor licensed psychotherapists. The final sample comprised *N* = 174 participants (81% female; *M*_age_ = 38.61, *SD*_age_ = 12.38; range: 23-76 years). Of them, 102 persons were licensed psychotherapists (58.6%), and 72 persons were psychotherapists-in-training (41.4%); all participants were psychologists. One-hundred sixty-two participants self-identified as cognitive-behavioral therapists (CBT, 93.1%), 15 as psychodynamic therapists (8.6%), eight as family therapists (4.6%), four as humanistic therapists (2.3%), and three as psychoanalytic therapists (1.7%). The majority of the participants worked in an outpatient setting (*n* = 125; 71.5%), 58 participants worked in an inpatient setting (33.3%), and 26 participants worked in a day-care setting (14.9%). Of note, some participants received training in more than one psychotherapeutic approach and/or worked in different settings. Therefore, the percentages do not add up to 100.

On average, participants treated two suicidal patients in the month prior to the survey (*M* = 1.83, *SD* = 3.53) and about six suicidal patients (*M* = 5.84, *SD* = 8.04) within the previous year. The mean work experience was 9.60 years (*SD* = 9.37; range: 1-48 years) and 66.1% stated that they knew the central recommendations of the German guideline on the treatment of unipolar depression.

### Procedure

An online survey questionnaire was composed with *SoSci Survey*. Social media postings were used to promote the study. The online survey was completed anonymously. Prior to assessments, participants were informed about the purpose of the study, the voluntary nature of their participation, data storage, and security. All participants gave their informed consent to participate via an online form. The Ethics Committee of the Ruhr-Universität Bochum approved the study (638/2020).

### Materials

Participants were presented with a total of five sets of questions, with a request to assess which recommendations for managing suicidal patients are provided in the German guideline for the treatment of depression. Within each question group, correct answers were supplemented by incorrect/distractor answer options (see Table 1).

**Table 1:** Overview of guideline recommendations and distractor items.

	**Recommendation**	**Correct answers**	**Incorrect answers**
1.	Inpatient admission should be strongly considered for suicidal patients, …	… who are acutely suicidal	… who show non-suicidal self-injury
		… who require medical care after a suicide attempt.	… who suffer from chronic suicide ideation.
		… who require intensive psychiatric or psychotherapeutic treatment because of the underlying depressive disorder.	… who show chronic parasuicidal behavior (e.g., driving without helmet).
		… if a sufficiently reliable assessment of suicidality is not possible in any other way.	… who suffer from long-lasting passive suicidal ideation.
		… if the establishment of a therapeutic relationship is not successful and the person remains acutely suicidal despite initial treatment.	
2.	For the specific acute treatment of suicidality, the following should be used/not used: …	Antidepressants should not be used.	Antidepressants, Ketamine, Benzodiazepine, Benzodiazepines in combination with Antidepressants, Electroconvulsive therapy should be used
3.	In relapse prophylaxis in suicidal patients, the following should be considered to reduce suicidal acts (suicide attempts and suicides): …	Lithium	Olanzapine, Carbamazepine, Lamotrigine
4.	Suicidal patients with a depressive episode should be offered …	… psychotherapy that initially focuses on suicidality.	… psychotherapy that focuses on the causing depressive disorder.
			… no psychotherapy, as it might have a destabilizing effect.
5.	A follow-up appointment of patients who were hospitalized for suicidality …	… should be scheduled in the short term, no more than one week after discharge because the risk for further suicidal acts is highest in the post-discharge period.	… should be scheduled within a month after discharge because the risk for further suicidal acts is highest in the post-discharge period.
			… should be scheduled within six months after discharge, because the risk for further suicidal acts is highest in the post-discharge period.
			… is not necessary, since the risk of suicidal acts is low after discharge.
			… is not necessary, when a medication is stably adjusted.
			… should be decided upon by an outpatient clinician.

Multiple answers were possible for three groups of questions (inpatient admission, antidepressant medication, lithium medication). Furthermore, participants could indicate that they did not know the answer. In addition, participants were asked how they themselves act in clinical practice. Since psychotherapists are not allowed to prescribe psychotropic drugs in Germany, the latter questions referred exclusively to the non-pharmacological treatment recommendations.

### Statistical Analyses

Data analysis was conducted using SPSS version 27.0 (14). Descriptive data are presented as frequencies (%) of correct answers. To explore differences between groups (psychotherapists in training vs. licensed psychotherapists) Chi-square tests were conducted. Bonferroni correction was used to adjust the probability value due to multiple testing. The *p*-value for all group comparisons was set at *p* < 0.005.

## Results

### Knowledge of Guideline Recommendations

**Figure 1:**
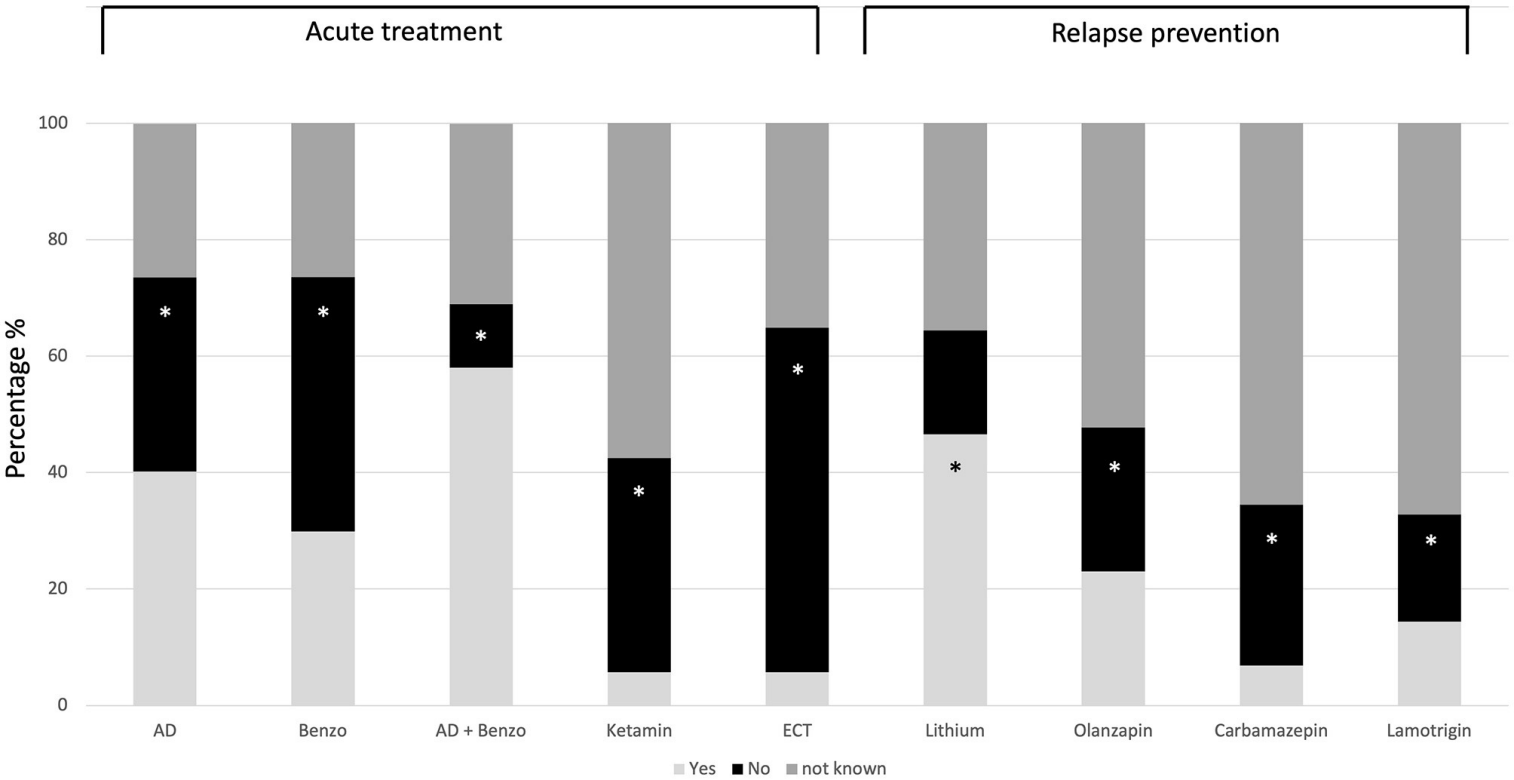
Response pattern to pharmacological treatment recommendations. The correct answer option is marked with a star (^*^). AD, Antidepressants; Benzo, Benzodiazepines; ECT, Electroconvulsive therapy; Yes, Should be used in treating suicidal patients; No, Should not be used in treating suicidal patients.

The percentages of correct identification of the guidelines recommendations on dealing with suicidal patients can be seen in Table 2, Figure 1. The rate of correct identification of guideline recommendations ranged from 33.3 to 95.4%.

**Table 2:** Percentages of consent to non-pharmacologic treatment recommendations.

**Questions and answers**	**Rate (%)**
**Inpatient admission should be strongly considered for suicidal patients**	
… who are acutely suicidal	**95.4**
… who require medical care after a suicide attempt	**64.9**
… who require intensive psychiatric or psychotherapeutic treatment because of the underlying depressive disorder	**46.0**
… if a sufficiently reliable assessment of suicidality is not possible in any other way	**82.2**
… if the establishment of a therapeutic relationship is not successful and the person remains acutely suicidal despite initial treatment	**84.5**
… who show non-suicidal self-injury	2.3
… who suffer from chronic suicide ideation	14.4
… who show chronic parasuicidal behavior (e.g., driving without helmet)	12.1
… who suffer from long-lasting passive suicidal ideation	7.5
**Suicidal patients with a depressive episode should be offered …**	
… psychotherapy that initially focuses on suicidality	**86.2**
… psychotherapy that focusses on the causing depressive disorder	7.5
… no psychotherapy, as it might have a destabilizing effect	0.6
**A follow-up appointment of patients who were hospitalized for suicidality**	
… should be scheduled in the short term, no more than one week after discharge, because the risk for further suicidal acts is highest in the postdischarge period	**40.8**
… should be scheduled within a month after discharge, because the risk for further suicidal acts is highest in the postdischarge period.	23.0
… should be scheduled within six months after discharge, because the risk for further suicidal acts is highest in the postdischarge period.	4.0
… is not necessary, since the risk of suicidal acts is low after discharge.	0.6
… is not necessary, when a medication is stably adjusted.	0.0
… should be decided upon by an outpatient clinician.	5.2

*The fields highlighted in gray refer to actual guideline recommendations*.

Mistakenly, 58% assumed that the guideline recommends a combination of antidepressants and benzodiazepines for the treatment of acute suicidal patients, 40.2% assumed that antidepressants should be used, 29.9% assumed that benzodiazepines should be used to treat acute suicidality, and 23% assumed that olanzapine should be used for relapse prophylaxis in depressed suicidal patients (see Figure 1). The rate of incorrect answers on the pharmacological treatment was below 14.4% with respect to all other questions. Yet, regarding pharmacological treatments between 26.4% (Benzodiazepine) and 67.2% (Lamotrigine) indicated that they did not know whether a given medication should be used or not (see Figure 1); 26.4% indicated that they did not know under which conditions a follow-up appointment should be scheduled, and 5.7% indicated that they did not know what kind of psychotherapy should be scheduled.

Taken together, the rate of those who answered all answer options on the question of acute pharmacological treatment correctly is 0.6% (*n* = 1). The rate of those who answered all questions about pharmacological relapse prevention correctly is 10.9 % (*n* = 19).

### Group Differences in Knowledge of Guideline Recommendations

Psychotherapists in training and licensed psychotherapists did not differ in identification correctness regarding inpatient admission, $\chi_{\left(1\right)}^{2}$ <6.13, all *ps* > 0.013; antidepressant medication, $\chi_{\left(1\right)}^{2}$ = 2.72, *p* = 0.099; lithium medication, $\chi_{\left(1\right)}^{2}$ = 0.82, *p* = 0.775; psychotherapy, $\chi_{\left(3\right)}^{2}$ = 9.47, *p* = 0.024 or follow-up appointments, $\chi_{\left(1\right)}^{2}$ = 13.99, *p* = 0.016.

### Adherence to Guideline Recommendations

In accordance with the guideline recommendations, 84.5% of the participants indicated that they themselves would offer psychotherapy that initially focuses on suicidality when dealing with suicidal depressed patients. Of those working in an inpatient setting or a day clinic, 13.2% indicated that they would offer a follow-up appointment 1 week after discharge to patients hospitalized due to suicidality. In their clinical practice, 99.4% would arrange an inpatient admission for patients who are acutely suicidal; 70.1% for patients who require medical care after a suicide attempt; 57.5% for patients who require intensive psychiatric or psychotherapeutic treatment because of the underlying depressive disorder; 86.8% for patients in case that a sufficiently reliable assessment of suicidality is not possible in any other way; and 87.4% in case that the establishment of a therapeutic relationship is not successful and the person remains acutely suicidal despite initial treatment. Less than 14.4% would arrange an inpatient admission due to one of the reasons given within the distractor items (e.g., chronic suicidal ideation, non-suicidal self-injury).

## Discussion

The present study investigated to what extent German psychotherapists are aware of the guideline recommendations (6) for the treatment of suicidal depressed patients. There are three main findings: (1) Knowledge and adherence to the guideline recommendations on the focus of psychotherapeutic treatment and the reasons for inpatient admission of suicidal patients were well present. (2) Knowledge about pharmacological treatment recommendations was low. (3) Knowledge on the necessity of immediate follow-up appointments after discharge of patients hospitalized due to suicidality was low.

In total, 33% of the respondents knew that antidepressants should not be used to treat acute suicidality according to the German guideline, and 0.6% of the participants were able to correctly answer all questions about pharmacological acute treatment. In meta-analytic reviews on randomized-placebo controlled trials, it has repeatedly been shown that antidepressants are not more effective than placebos in preventing suicide attempts and suicides (15–18), with some meta-analysis pointing to a higher rate of suicide attempts and suicides in patients being treated with antidepressants compared to patients receiving placebo [e.g., (19)]. In addition, antidepressant prescribing rates do not appear to be associated with population-based suicide rates (20, 21). The question arises as to why the belief in an antisuicidal effect of antidepressants continues to persist: On the one hand, it is known that placebo does have an effect (22), so it is likely that those treated with antidepressants experience psychological stabilization (and practitioners thus experience the medication as helpful). On the other hand, suicide attempts and suicides are very rare even under antidepressant treatment (19), so practitioners would have to treat an extremely large number of patients to be able to perceive a negative effect of antidepressants at all. These aspects may contribute to the fact that comparatively few of the psychotherapists interviewed were aware of the lack of effect of antidepressants. In addition, many participants (58%) assumed that the guideline recommends a combination of benzodiazepines and antidepressants in the treatment of suicidal patients. In fact, there are references to this in the literature (23). But since there is no strong empirical evidence for the benefit of such a treatment strategy, it is not a guideline recommendation. According to the guideline, benzodiazepine mono-treatment can be used for a maximum period of 14 days. However, since there is also no strong empirical evidence for this form of treatment, the guideline “allows” the usage of benzodiazepines in the treatment of suicidal patients (by stating “benzodiazepines *can* be used”), but gives no firm recommendation for this kind of treatment (i.e., the guideline does not say “benzodiazepines *should* be used”). Treatment with Ketamine was not yet described in the 2015 guideline. In the meantime, there are studies that point to a rapid antisuicidal effect of ketamine (24), but since the study situation is still heterogeneous and characterized by methodological deficiencies (25), no strong recommendation for Ketamine treatment will be made in the soon to be published 2022 updated version of the German guideline on the treatment of unipolar depression. The only drug recommended in the guideline is lithium, as an antisuicidal effect of lithium could be proven in studies (26). Of the study participants, 46.6% knew this. No recommendations are available for other phase prophylactics. Notably, German psychotherapists are not allowed to prescribe medications. Nevertheless, psychotherapists need to know about the indications for groups of medications. They must be able to explain alternative treatment options and coordinate the involvement of psychiatrists, neurologists, and other mental health workers. On this background, the largely inadequate knowledge of drug treatment options for suicidal patients is a cause for concern.

This is equally true with respect to the low level of knowledge about the need to schedule a follow-up appointment within 1 week of inpatient discharge with patients hospitalized for suicidality. Of those surveyed, about 41% knew that such a procedure was recommended by the guideline, but only 13% of those working in an inpatient or day hospital setting reported offering such follow-up appointments. It can be assumed that follow-up appointments are not part of the treatment routine in many hospitals. In view of the fact that the suicide rate after discharge from an inpatient treatment is significantly increased (27) and that in other medical disciplines outpatient follow-up appointments are part of the standard of care (e.g., after a heart attack), it is incomprehensible why corresponding services are not routinely established in psychiatric settings as well. In conclusion, the findings point to the need for greater dissemination of various facts about the management of suicidal patients.

On the other hand, knowledge about the conditions under which inpatient admission is appropriate and the fact that psychotherapy should initially focus on suicidality is widely present. Still, it is unclear to what extent suicide-specific psychotherapy programs (28–30) are actually known and used. In addition, the reasons for inpatient admission are less empirical than consensual in nature, so deviations from the guideline are neither surprising nor alarming.

In general, suicide prevention training has been shown to improve the knowledge, skills, and attitudes toward suicidal behavior of mental health professionals [cf., (31, 32)]. More specifically, de Beurs et al. (33) found that an e-learning supported Train-the-Trainer program is an effective strategy for implementing clinical guidelines for suicidal patients. In this multicentre cluster randomized controlled trial focusing on mental healthcare departments throughout The Netherlands, medical staff (nurses, psychologists, and psychiatrists) either received a 1-day interactive group program supported by e-learning modules in which the content of The Netherlands practice guidelines for the assessment and treatment of suicidal behavior was taught, or they received no training. At a 3-month follow-up, professionals who received the intervention showed greater guideline adherence than professionals who were only exposed to traditional guideline dissemination (that is dissemination via websites, presentations at conferences, books, manuals), but received no specific training (33). Although the training intervention has had no effect on patients' suicidal ideation and suicidal behavior (34), a subgroup analysis showed that patients who were suicidal and suffered from depression showed a significant decrease in suicide ideation when treated by professionals of the training group [cf., (35)]. Thus, both professionals and patients benefited from appropriate training. In Germany, such systematic training on the assessment and treatment of suicidal patients has not yet been offered and studied. However, the available findings indicate that this might be warranted.

Some limitations have to be considered when interpreting the current results. First, the study focused exclusively on German psychotherapists. Therefore, it is unclear in as much the findings generalize to other mental health professions. In this context, it would be particularly interesting to assess the level of knowledge and adherence of psychiatrists, psychiatric nurses and social workers employed in an inpatient psychiatric setting. With regard to the guideline recommendations on pharmacological treatment, it is especially relevant to assess the level of knowledge and adherence of physicians, since in Germany only physicians are allowed to administer medication. Nevertheless and as already mentioned, the basic guideline recommendations should be known to all mental health professionals. Second, the study assessed only self-reported adherence to the guideline recommendations. A follow-up study should therefore investigate the extent to which the guideline recommendations in dealing with suicidal patients are actually taken into account in practical work and how this, in turn, affects the effectiveness of treatment [cf., (34)]. Third, not all recommendations of the guideline on the management of suicidal patients were taken into account in the current study, but only those with a strong recommendation level, i.e., recommendations that are not only based on a consensus decision but on empirical data. Furthermore, no recommendations on the management of suicidal patients from guidelines on other mental disorders were considered in the present study. In a future study, therefore, a more comprehensive assessment of knowledge and adherence to the management of suicidal patients should be carried out. Finally, the guideline recommendations used within this study are from the 2015 version of the German guideline for the treatment of unipolar depression (6) and not from the updated version of the guideline that will be published in 2022. However, the guideline recommendations will remain largely unchanged in the updated version. Accordingly, the deficiencies found here would remain valid on the basis of the updated guideline.

## Conclusion

Taken together, the study results indicate that some recommendations of the German guideline for the treatment of depressed suicidal patients are not well-known. This applies in particular to pharmacological treatment and follow-up after inpatient therapy. The knowledge of effective and less effective methods of suicide prevention needs further dissemination. Specific training programs modeled after the Train-the-Trainer intervention by de Beurs et al. (33, 34) could be used for this purpose.

## Data Availability Statement

The raw data supporting the conclusions of this article will be made available by the authors, without undue reservation.

## Ethics Statement

The studies involving human participants were reviewed and approved by Ethics Committee of the Faculty of Psychology, Ruhr-Universität Bochum, Germany. The patients/participants provided their written informed consent to participate in this study.

## Author Contributions

TT: conceptualization, methodology, and writing of the original draft and editing. HD and LE: investigation and data curation. TT, JB, and JC: formal analysis and revising of the manuscript. All authors contributed to the article and approved the submitted version.

## Conflict of Interest

The authors declare that the research was conducted in the absence of any commercial or financial relationships that could be construed as a potential conflict of interest.

## Publisher's Note

All claims expressed in this article are solely those of the authors and do not necessarily represent those of their affiliated organizations, or those of the publisher, the editors and the reviewers. Any product that may be evaluated in this article, or claim that may be made by its manufacturer, is not guaranteed or endorsed by the publisher.
